# CC Chemokine Ligand 20 and Its Cognate Receptor CCR6 in Mucosal T Cell Immunology and Inflammatory Bowel Disease: Odd Couple or Axis of Evil?

**DOI:** 10.3389/fimmu.2013.00194

**Published:** 2013-07-15

**Authors:** Adrian Y. S. Lee, Rajaraman Eri, Alan B. Lyons, Michael C. Grimm, Heinrich Korner

**Affiliations:** ^1^Menzies Research Institute Tasmania, University of Tasmania, Hobart, TAS, Australia; ^2^School of Medicine, University of Tasmania, Hobart, TAS, Australia; ^3^School of Human Life Sciences, University of Tasmania, Launceston, TAS, Australia; ^4^St. George Clinical School, University of New South Wales, Sydney, NSW, Australia

**Keywords:** inflammatory bowel diseases, Th17 cells, T_reg_ cells, CCR6, CCL20

## Abstract

Chemokines and their cognate receptors have been identified as major factors initiating and governing cell movement and interaction. These ligands and their receptors are expressed on a wide variety of cells and act during steady-state migration as well as inflammatory recruitment. CCR6 is a non-promiscuous chemokine receptor that has only one known chemokine ligand, CCL20, and is present on B and T cells as well as dendritic cells (DCs). Two CD4^+^ T cell populations with opposing functions present in the intestines and the mesenteric lymph nodes express CCR6: the pro-inflammatory T_H_17 and regulatory T_reg_ cells. CCL20 is also present in the intestine and is strongly up-regulated after an inflammatory stimulus. Interestingly, this ligand is also expressed by T_H_17 cells, which opens up the possibility of autocrine/paracrine signaling and, consequently, a self-perpetuating cycle of recruitment, thereby promoting inflammation. Recently, CCR6 has been implicated in inflammatory bowel disease (IBD) by genome wide association studies which showed an association between SNPs in the genomic region of the CCR6 gene and the inflammation. Furthermore, recent research targeting the biological function of CCR6 indicates a significant role for this chemokine receptor in the development of chronic IBD. It is therefore possible that IBD is facilitated by a disordered regulation of T_H_17 and T_reg_ cells due to a disruption in the CCL20-CCR6 axis and consequently disturbed mucosal homeostasis. This review will summarize the literature on CCL20-CCR6 in mucosal immunology and will analyze the role this receptor-ligand axis has in chronic IBD.

## Chemokines: Biochemistry and Function

The chemokine family is a large collection of positively charged small cytokines that have been shown to be important cues in the chemotactic navigation of leukocytes to sites of inflammation (1, 2). In addition, they are recognized as molecules central for the normal development of lymphoid tissues, immune cell maturation/development, and immunological homeostasis (3).

Chemokines have a highly conserved, unique structure that has allowed a comprehensive *in silico* detection of characteristic patterns and a consistent classification in subfamilies (4). Four conserved signature cysteine residues linked by one to three disulfide bonds typically characterize the molecules. Depending on the position of two of the conserved cysteines near the *N*-terminus, they are categorized as CC (Cysteine–Cysteine; cysteines adjacent to each other) or CXC [cysteines separated by one non-conserved amino acid (X)] chemokines. These subfamilies are also known as β- and α-chemokines, respectively. Two further smaller classes of chemokines termed C (γ−) and CX_3_C (δ−) chemokines exist with only two and one members, respectively (4).

Chemokines are usually secreted from the cell after their synthesis; but may become tethered to glycosaminoglycans (GAGs), a family of sulfated polysaccharides which can be located in the extracellular matrix or on the extracellular surface (5), or other sulfated sugars to form a stable local chemokine gradient and support the binding of a cognate chemokine receptor. This form of “haptotaxis,” the movement along stable, immobilized gradients, has been described as the most likely physiological form (6) while gradients relying on soluble chemokines can only be envisaged as transient and short-lived in response to a local inflammatory stimulus.

The classical chemokine receptor has seven hydrophobic transmembrane domains arranged as α-helices, an extracellular *N*-terminus and intracellular *C*-terminus (7). After binding to the cognate partner, signaling is transmitted by the G-proteins (8). Following ligand binding, the receptor is rapidly internalized, either via clathrin-mediated or caveolae-dependent endocytosis, in a process known as homologous desensitization, which protects the cell from over-stimulation (9). In addition, chemokine receptors are biochemically and functionally regulated by non-chemokine ligands interacting with their own cognate receptors (e.g., opioids/opioid receptors, vasoactive intestinal peptide, and its receptors); a process known as heterologous desensitization (10, 11).

Chemokine genes tend to be genomically clustered, with human CXC chemokines located around chromosome 4q12–13 and CC chemokines around chromosome 17q11.2. Each main grouping tend to promiscuously share receptors of the same subtype (4).

## The CCL20-CCR6 Axis

The cysteine–cysteine motif chemokine ligand 20 (CCL20) – also known as liver- and activation-regulated chemokine (LARC), macrophage inflammatory protein-3α (MIP-3α), and exodus-1 – was discovered independently by three research groups using bioinformatics techniques (12–14). The chemokine CCL20 is encoded by the *SCYA20* [small inducible cytokine family A (Cys–Cys), member 20] gene (4). Unlike other CC chemokines, *SCYA20* was localized to chromosome 2q33–q372 using a combination of PCR analysis of single chromosome DNA hybrids, yeast artificial chromosome clones, and radiation hybrid mapping (4, 14). The full length of human CCL20 cDNA contains 799 bp (4 exons and 3 introns), encoding an open reading frame of 95 or 96 AA (14–16). Since the putative cleavage site of this precursor protein is between Ala-26 and Ala-27, the mature form is a polypeptide with a length of 70 AA (14). Interestingly, two variants of CCL20 cDNA were detected in which the shorter, truncated isoform had a deletion of three base pairs [one amino acid (Ala-27, GCA)] at the *N*-terminus (15). As a result, the two forms, which probably are the consequence of allelic polymorphism and use of different splicing sites, have been termed the Ala-27 and Ser-27 forms of CCL20. However, examination of the biological activity through chemotaxis assays of both revealed only a negligible difference in chemotactic profiles (15).

CCL20 is expressed in a variety of human tissues and by a range of immune cells. Based primarily on early expression assays utilizing Northern blots to detect CCL20 mRNA, constitutive expression was ascertained to be predominantly in human organ-associated lymphoid tissue (lungs, lymph nodes, appendix, etc.), epithelial cells (including the intestines), and the liver; but surprisingly, virtually non-existent in spleen or bone marrow (12, 14, 16, 17). The presence of CCL20 in these tissues where active sites of inflammation and immune activation occur suggested that CCL20 was, to some extent, an inflammatory chemokine (12). Indeed, inflammation-related cells which have been shown to secrete or express CCL20 include endothelial cells (18, 19), neutrophils (20), natural killer (NK) cells (21), T_H_17 cells (22), B cells (23), and a variety of other immune cells [dendritic cells, DCs, Langerhan’s cells (LCs), and macrophages] (13, 24).

## The Cognate Receptor of CCL20: CCR6

As a unique feature among the CC chemokine family and consistent with its location on chromosome 2, CCL20 has a sole known receptor, CCR6 (25–27), and their interaction has been confirmed when CCL20 was able to induce calcium mobilization in K562 cells transfected with CCR6; but not with any other chemokine receptor CCR1–5 (27). In addition, 5 other CC chemokines tested (CCL2–5 and CCL17) failed to bind to CCR6 (27).

Upon binding of CCL20 to CCR6, the receptor is internalized, exhibiting decreased cell surface expression of CCR6 on splenocytes and especially on B cells (18). The receptor is found on a number of different immune cells, including CD34^+^ hematopoietic precursor-derived DCs (28), memory T cells (29), and peripheral and memory B cells (18, 23, 30). Early investigations into tissue expression were performed by Northern blot analysis, revealing significant presence in human liver, appendix, and lymph nodes, and minor presence in the thymus, testis, and small intestine (27). CCR6 mRNA is up-regulated in T cells by inflammatory stimuli (27). In B cells, it undergoes a series of up- and down-regulation during B cell development and activation (23, 31, 32). This suggests that CCR6 is involved in both inflammatory and homeostatic roles in the immune system (33).

## CCL20 and its Receptor in an Inflammatory Environment

The up-regulation of CCL20 in inflammation has been documented well in the literature. Under experimental conditions, various cytokines (IL-1α, IL-β, IL-17, IL-21, IFN-γ, TNF-α) have been found to induce CCL20 expression at various micro-anatomical locations (34–36). In contrast, IL-4, IL-22, and IL-23 have been reported to have a negligible inductive effect on CCL20 (34, 36) and application of the anti-inflammatory cytokine IL-10 down-regulates CCL20 expression (12) as does the addition of IFN-γ in synoviocytes (36). By utilizing CCL20 promoter region luciferase-reporter constructs transfected into HEK293T, Caco-2, and G-361 cell lines, researchers identified a NF-κB site upstream from the transcription start site that was, at least in part, responsible for the inflammatory responsiveness of CCL20 to TNF-α and IL-β induction (37–39).

## Role of CCL20-CCR6 Axis in Development of the Intestinal Immune System

Northern blot analysis of CCL20 expression shows high constitutive mRNA expression at mucosal sites such as the intestines, while CCR6 mRNA is only expressed at low to moderate levels (27). In a more detailed analysis of murine Peyer’s patches (PP), CCL20 mRNA was found to be concentrated in the overlying follicle associated epithelium (FAE) whilst CCR6 mRNA could be detected in the sub-epithelial dome (SED) on CD11b^+^CD11c^+^CD8a^−^myeloid DCs (40). The latter expression was confirmed later on protein level in a study using an eGFP-CCR6 knock-in mouse strain (41). Furthermore, in the absence of CCR6, PP were smaller than their WT counterparts, yet were unchanged in absolute numbers and in proportions of lymphocytic subpopulations (42). Interestingly, intraepithelial lymphocytes were proportionally increased when compared to WT mice (42).

In a different study, a substantial expression of CCL20 was detected in isolated lymphoid follicles (ILF) and a number of developmental problems were noted in the intestinal lymphoid tissues of CCR6-deficient mice such as smaller PP displaying a reduced number of domes, and the absence of ILF (43). As a consequence of these immunological deficiencies, CCR6^−/−^ mice show a diminished IgA response to rotavirus (33). Furthermore, it has been shown that the absence of CCR6 reduced intestinal M cell numbers (44, 45). Interestingly, because the bacteria *Yersinia enterocolitica* utilize M cells to cross the intestinal lumen, CCR6-deficient mice have increased resistance to this microorganism compared to WT mice (46). Collectively, the above results point to a role of the CCL20-CCR6 axis in structural and functional aspects of the mucosal immune system of the intestines.

## Defensins

A curious aspect of the CCL20-CCR6 axis in mucosal immunity is the relationship between CCL20 and β-defensins, small anti-microbial peptides found in epithelial tissue and at mucosal surfaces after inflammatory stimulus such as TNF (47, 48). The human polypeptide β-defensin (type 1 and 2) is able to bind to CCR6 – potentially being capable of displacing CCL20 from its receptor – and hence act as a chemoattractant for CCR6-bearing immature DCs and various T cell subsets (49). This behavior can be explained by the structural similarities between defensins and CCL20. The two β-defensins each have a α-helix and three-stranded β-sheets at their *N*-termini in an arrangement similar to human CCL20. In turn CCL20 is able to display direct anti-microbial activity *in vitro* (50). However, this aspect in CCL20-CCR6 immunobiology has been challenged in recent years and inconsistencies in the literature exist. Whilst human β-defensins (hBDs) were chemotactic for macrophages, CCL20 failed to elicit such migration *in vivo* or *in vitro* (51). This suggests that another yet-to-be-identified receptor for β-defensins exists for chemoattraction. Indeed, a more recent study found that hBD-2 and -3 attracted human monocytes through interaction with CCR2 (52) and hence, this raises a possible alternative to the β-defensin-CCR6 relationship (53).

## Biology of IBD and Mouse Colitis Models

Crohn’s disease (CD) and ulcerative colitis (UC) are the two dominant subtypes of inflammatory bowel disease (IBD). Both are chronic inflammatory diseases of the intestinal tract; but while CD can involve any part of the intestinal system and presents with a transmural chronic inflammation spanning the entire intestinal wall, UC is limited to the colon, commencing distally, and the inflammation is confined to the mucosa and submucosa. The epidemiology of IBD shows a strong genetic predisposition for this chronic immune response, compounded by environmental factors such as microbiota and diet.

In humans, genome wide association studies (GWAS) have identified a plethora of genetic loci associated with IBD (54–57). These loci include genes that are associated with innate immune responses, such as *NOD2*, autophagy, such as *ATG16L1* and with regulation of leukocyte migration such as the chemokine receptor *CCR6* (55). This indicates that both regulatory and functional variations are associated with the generation of IBD. Furthermore, it has been demonstrated that CCL20 expression is up-regulated in human IBD biopsies predominately associated with FAE in a TNF-dependent manner, thus indicating a potential location for interaction between CCR6^+^ T cell subsets and DCs (58). Mouse models of acute colonic inflammation, however, are conflicting in their results. In the dextran sulfate sodium (DSS) model of colitis, CCR6^−/−^ mice developed less severe colitis compared to WT mice (59), while in the 2,4,6-trinitrobenzene sulfonic acid (TNBS)-induced model, the clinical picture of colitis was stronger (60). In a third widely used classical model of colitis induced by T cell transfer, naïve T cells from CCR6^−/−^ mice transferred into *Rag2*^−/−^mice caused a very severe colitis compared to CCR6^+/+^ transferred T cells. In this model, CCR6^−/−^ T_reg_ cells displayed less suppressive capabilities and reduced migration to the site of inflammation compared to WT T_reg_ cells. This indicates that the protection from colitis is offered by a novel colon-homing, IL-10-producing CCR6^+^ regulatory T cell population (61). These results are in part explained by a recent report on the generation and education of a T_H_17 subset in the gut, which provided an interesting insight into the regulation of these inflammatory T cells (62, 63). Using a T cell-depletion model of colitis, it was demonstrated that T_H_17 cells accumulated in great numbers in the small intestine when a strong T cell receptor stimulus was present. More importantly, it was established that this T_H_17 accumulation was dependent on the CCL20-CCR6 axis and that these T_H_17 cells, interestingly, acquired an immunosuppressive phenotype in the gut, rather than the typical inflammatory phenotype associated with this cell subset (62). This was determined when the accumulated T_H_17 cells were able to suppress CFSE-labeled responder T cells (CD4^+^CD25^−^) in a suppression assay (*in vitro*) and prevent the establishment of EAE in experimental transfer models (*in vivo*). The likely mechanism of these cells is through the secretion of the immunosuppressive cytokine IL-10 (62).

## CCL20-CCR6 in Mucosal T_H_17 and T_reg_ Biology

The role that the CCL20-CCR6 axis has to play in steady-state dynamics is currently unclear; but the fact that opposing cell subtypes (T_H_17 and T_reg_ cells) express and respond to CCL20 hints at a potential regulatory balance between immune activation and suppression, and implies an intriguing feedback loop (64). Already, CCL20 has been implicated in a network of effector and regulatory immune functions through the finding of CCR6 on the opposing T_H_17 and T_reg_ cells and CCL20 secretion from the former (22). Supporting the axis’ functionality, experimental models of pathology have demonstrated the necessity for CCR6 in the recruitment of these cell types to the sites of inflammation following an up-regulation of CCL20 (65). In a *Rag1*^−/−^ severe combined immunodeficiency (SCID) model where CCR6^−/−^ or WT T_H_17 cells were transferred in, a reduction in both T_H_17 and T_reg_ cells were seen in the recipient mice receiving the CCR6^−/−^ cells. This, interestingly, resulted in more severe colitis in these animals (66). Paradoxically, mice deficient in CCR6 had an attenuated course of inflammation in an experimental autoimmune encephalomyelitis (EAE) model of disease (67). Collectively, these findings highlight the dynamic balance that exists between T_H_17 and T_reg_ cells and how the chemokine axis may be fundamental to ascertaining in which direction the balance is tipped. Some studies using CCR6^−/−^ mice seem to imply that CCR6 is functionally more important to T_reg_ cells than it is to T_H_17, resulting in more severe pathology in these knockout mice (61, 68).

Examination of organs of 5- to 7-week-old naive BALB/c mice determined that most T_H_17 cells are found in small intestine, PP, and colon at immunologic steady-state. In contrast, the T_reg_ cells are predominantly found in the bone marrow, colon, and peritoneal cavity (66). Interestingly, it appears that CCR6 is a dominant receptor for the migration of T_H_17 cells to PP (but not of T_regs_). This was supported through a later study that found the CCL20-CCR6 axis helps direct T_H_17 cells to the small intestine upon immune system induction. Furthermore, IL-17 secreted from T_H_17 cells up-regulates intestinal epithelial CCL20 which helps recruit these cells via the CCL20-CCR6 axis. Curiously, the CCR6-deficient mice had increased T_H_17 cells in the spleen and lymph nodes, suggesting that the effector cells are unable to migrate from the sites of induction. This resulted in less intestinal inflammation in these knockout mice as expected (62).

Quite a large number of effector T_H_17 cells can be found in the small intestine under steady-state conditions and during inflammation, compared to peripheral sites (62, 69). The precise roles that T_H_17 cells and their cytokines play in mucosal immunity have been the subject of a plethora of ongoing investigations. T_H_17 cells seem particularly important in mediating normal defense at mucosal sites, as demonstrated in T_H_17- and IL-17-deficient mice that showed marked susceptibility to oral *Candidiasis* compared to WT counterparts (70). Additionally, in induced immunodeficiency mice models, the absence of T_H_17 cells allowed a greater dissemination of *Salmonella typhimurium*, from the gut to other parts of the body (71). Indeed, the means of protection under steady-state conditions appear to be through the cells’ ability to organize the immunological and microbiological barriers against potentially harmful pathogens (72). Therefore, the CCL20-CCR6 axis, which appears to be integral to T_H_17 homing and function, is important for steady-state mucosal immunity in the gut.

The microbiological environment of the small intestines has been shown to have a dominant reciprocal effect on T_H_17 cell quantity with studies showing that intestinal microbiota are necessary for steady-state colonization and maintenance of the cells (73, 74). In support of this observation, mice treated with antibiotics had their intestinal T_H_17 cell population essentially ablated (73). In addition, mice that were housed in a germ-free environment – therefore lacking intestinal microbiota – not only had reduced T_H_17 colonization but had a reciprocal increase in the FoxP3^+^ regulatory T cells suggesting that the microbiota influences the T_H_17-T_reg_ balance in the gut (74). Finally, the colonization of the intestines by the T_H_17 subset is also functionally necessary as it is associated with protection against intestinal pathogens such as *Citrobacter rodentium* (73). Mice deficient for the T_H_17 subset-associated pro-inflammatory cytokine IL-21 were consequently protected against the development of DSS colitis and failed to up-regulate other critical T_H_17-associated molecules such as IL-17 and ROR-γt, which could be confirmed in the TNBS-induced colitis model (75).

These findings imply that the CCL20-CCR6 axis, in conjunction with the microbiota, have dominant effects on regulating the T_H_17-T_reg_ balance and are therefore influencing the pathogenesis of colitis. Future studies to explore this aspect can employ the use of other mouse models to further clarify the relationship between CCR6, T_H_17 cells, and colitis. One such model is the so-called Winnie mouse strain (76, 77), which is a model of IBD, based on a single point mutation of the *Muc2* mucin gene generated by ENU mutagenesis. This mutation results in a proportion of the MUC2 protein misfolding and accumulating in the endoplasmic reticulum (ER) resulting in ER stress in intestinal goblet cells, a depleted intestinal mucus barrier and spontaneous colonic inflammation (78). The distal colon segment showed significant increase in the expression of T_H_17 signature genes; *Il17a, Il17f, Tgfß*, and *Ccr6* were detectable suggesting a T_H_17 polarization. Hence, this novel model is ideally suited to study the role of CCR6 and T_H_17 cells in intestinal immune responses.

## Conclusion

The unique CCL20-CCR6 axis poses as a dilemma for researchers into IBD and other diseases. There is little doubt that the pair is involved in mucosal immunity and IBD; but because of its participation in the opposing T_H_17 and T_reg_ cell activities as well as B cell activation and antigen presentation, ascertaining its overall involvement in pro- or anti-inflammatory activities poses some difficulties. The differing approaches and experimental techniques may account for some of the contradictions in the above results of the precise contributions of the CCL20-CCR6 pair to IBD. A fundamental problem is that many studies rely on well established but not tissue-specific *Ccr6* gene knockout mice while CCL20^−/−^ mice do not yet exist. Therefore, unanswered questions remain: is the CCL20-CCR6 axis centrally involved in the development of inflammatory diseases of the bowel (axis of evil) or is it just a pair of cellular navigational tools that seemingly interfere with disease pathogenesis as an epiphenomenal artifact of a general knockout mouse (odd couple)? This question can be answered when better tools such as tissue-specific knockouts are available. Furthermore, we have to wonder how the axis can contribute to inflammatory cell recruitment yet confer suppression at the same time through the opposing T_H_17 and T_reg_ cells. Adding to the confusion, what role, if any, does the axis play in regulating the inflammatory versus regulatory T_H_17 cells? The answer to this question and others may lie in the combination of other regulatory factors, such as other chemokines, which recruit and “switch on” pro- or anti-inflammatory cells during IBD and will have to be addressed and dissected in more detail in further studies.

## Conflict of Interest Statement

The authors declare that the research was conducted in the absence of any commercial or financial relationships that could be construed as a potential conflict of interest.
